# Environmental health surveillance in a future European health information system

**DOI:** 10.1186/s13690-018-0272-6

**Published:** 2018-06-28

**Authors:** Anke Joas, Miriam Schöpel, Madlen David, Maribel Casas, Gudrun Koppen, Marta Esteban, Lisbeth E. Knudsen, Martine Vrijheid, Greet Schoeters, Argelia Castaño Calvo, Gerda Schwedler, Marike Kolossa-Gehring, Reinhard Joas

**Affiliations:** 1 BiPRO GmbH, Werinherstr. 79, 81541 Munich, Germany; 2 German Environment Agency, Berlin, Germany; 3 Flemish Institute for Technological Research, Mol, Belgium; 4 CNSA - ISCIII, National Centre for Environmental Health, Instituto de Salud Carlos III, Madrid, Spain; 5 ISGlobal, Barcelona, Spain; 6 Universitat Pompeu Fabra (UPF), Barcelona, Spain; 7 Spanish Consortium for Research on Epidemiology and Public Health (CIBERESP), Valencia, Spain; 8 University of Copenhagen, Copenhagen, Denmark

**Keywords:** HBM, HES, Health information, Environmental health, Birth cohorts, Chemical contaminants

## Abstract

**Background:**

To date Health information (HI) in the European Union does not comprise indicators or other information related to impacts of hazardous chemicals in consumer products, food, drinking water or air on the health status of the population. Therefore, we inventorised and evaluated the potential of environmental health surveillance and research data sources in the European population to provide HBM-based indicators of internal human exposure and health impact of relevant chemicals.

**Methods:**

We established an up-dated inventory of European cross-sectional Human Biomonitoring (HBM) surveys and of birth cohorts, and compared chemicals and chemical groups addressed by HBM with indicators and health end points collected via European Core Health Indicators (ECHI), in birth registries, as well as in environmental and food data bases and health registries to see on how data collection could be aligned. Finally, we investigated study designs of HBM survey and health examination surveys for potential synergies.

**Results:**

The inventory covers a total of 11 European cross-sectional national programmes and a large number of birth cohorts and includes information on study population, age groups, covered substances, sampled matrices, and frequency. The comparison of data collections shows that there are many overlaps between environmental chemicals with environmental and health reporting. HBM data could be linked with ECHI indicators for work-related risks, body mass index (BMI), and low birth weight, with perinatal disease, neurologic disorders, and some chronic diseases, or with data bases for e.g. indoor air, food, or consumer products. Existing initiatives to link data collections at European Environment Agency (EEA) and Joint Research Center (JRC) or at World Health Organization (WHO) are good options to further develop linkage of HBM with exposures sources and health end points.

**Conclusions:**

There is potential to use HBM based information in a number of public health policies, and this would help to align reporting to international commitments. Environmental health surveillance based on HBM and HBM-based indicators, is an excellent tool to inform public health policies about risks from environmental chemicals, and the EU health information system would benefit from additional HBM-based indicators for monitoring exposure burden from environmental chemicals. Considerable efforts are needed to align and establish routine data collections and to develop a surveillance system and indicators which may inform public health policies.

**Electronic supplementary material:**

The online version of this article (10.1186/s13690-018-0272-6) contains supplementary material, which is available to authorized users.

## Background

The Public Health Programme asks for better information on determinants for health, and the 2030 Agenda for Sustainable Development stresses the need to protect citizens from hazardous chemicals, as the target 3.9 of the 2030 Agenda asks to substantially reduce the number of deaths and illnesses from hazardous chemicals in air, water and soil pollution and contamination.

Furthermore, the Public Health Programme aims at preventing diseases and foster supportive environments for a healthy lifestyle, and to protect European Union citizens from serious cross-border health threats, taking into consideration social and gender specific differences.

The term “Health information” (HI) compromises information, data, and evidence that describe the health status of a population or a specific population group whereby the information is regularly expressed in forms of indicators. Health indicators describe health status, determinants of health, and health care. They allow monitoring and comparison, and serve as a basis for policy-making. To date HI in the European Union only comprises very few indicators related to internal human exposure and health impact of tobacco smoke and particulate matter but does not include any indicator related to other hazardous chemicals in consumer products, food, drinking water or indoor air.

European indicators in the field of environmental health are limited to a few indicators in the WHO European Region Environment and health information system (ENHIS^1^) list and in the European Core Health Indicators (ECHI^2^) list [1].

ENHIS comprises levels of lead in children’s blood, persistent organic pollutants (POPs) in human milk, exposure to air pollution (particulate matter, PM) in outdoor air, exposure of children to chemical hazards in food, and exposure of children to second-hand tobacco smoke (SHS).

Currently there are only two European indicators (assessing the exposure to PM 2.5, PM 10 and tobacco smoke) assessing impacts of environmental chemicals on health based on HBM.

From the 88 established ECHI indicators only a few ECHIs relate to environmental health such as smoking, consumption of fruit and vegetables, work-related health risks (such as COPD, injuries at workplace, percentage of employees who think their health is negatively affected by their work) and PM exposure. There is no indicator assessing impacts of environmental or occupational chemicals on health in ECHI yet.

The European Environmental Agency (EEA) gather information on pollutants in air, soil, water (Environmental Core Indicators) and the European Food Safety Agency (EFSA) collects information on intake and contamination of food.

Human Biomonitoring (HBM) is a well-established worldwide-applied methodology to assess whether and to what extent substances have entered the body. By measuring the concentrations of natural and synthetic compounds in body fluids (e.g. blood, urine, and breast milk) or tissues (e.g. hair, nails, fat, and bone), HBM can provide information on occupational or environmental exposures and, help identifying potential health risks.

HBM assesses the exposure in an integrated way considering all exposure pathways (inhalative, dermal, and/or oral) and sources, and the individual susceptibility and/or physiological status.

HBM studies are useful tools to elucidate associations between exposure burden and health endpoints. However, it is important to note that biomonitoring must be integrated with environmental monitoring, toxicological data and especially with the epidemiological considerations as highlighted by the European commission in the European Environment and Health Action Plan for 2004–2010 [2].

A major European initiatives to foster use of HBM in HI and policy making were the ‘Consortium to Perform Human Biomonitoring on a European Scale‘(COPHES/DEMOCOPHES)^3^ that established a European wide protocol and results for plasticizers (phthalates, bisphenol A) and cotinine and cadmium in urine, and mercury in hair [3, 4]. The project was funded under the 7th Framework Programme for Research and Technological development, as well as the FP7 funded frameworks ‘Developing a Child Cohort Research Strategy for Europe’ (CHICOS) and ‘Environmental Health Risks in European Birth Cohorts’ (ENRIECO).^4^ CHICOS identified all European birth cohorts [5] and ENRIECO specifically summarized info of those cohorts that have collected data on environmental exposures [6]. The ENRIECO inventory has facilitated the development of many collaborative studies pooling data across birth cohorts with the objective to examine the link between environmental exposures and health outcomes [7–9].

This publication serves, within the framework of the EU funded BRIDGE-Health project, as a comprehensive evaluation on i) the data available on environmental health surveillance in Europe, mainly coming from cross-sectional and birth cohort studies and ii) the similarities and differences between health examination studies (HES), health register information, and human biomonitoring (HBM). This overview was prepared with the aim to have a better integration of environmental health surveillance into HI and public health policies in the EU. This aim is also pursued by the European Union in the context of the Health in all Policies (HiAP) strategy which stresses, among others, that health is determined by the conditions in which people live, work, and play. This approach is also acknowledged in the coming years, when European HBM is continued in an H2020 funded joint programme “HBM4EU”.^5^ In this context, this publication will be of fundamental importance, as the HBM4EU project also aims at improving the integration of HBM and health studies data.

## Methods

We established an up-to-date inventory of chemicals and other environmental risk factors assessed by means of cross-sectional HBM studies, and birth cohorts. The inventory contains information about study objectives and collected data (analysed chemicals, matrix, sociodemographic characteristics of participants, etc.). The sociodemographic characteristics of participants, and the scientific focus of each study were summarized. The database is easily accessible, and allows to see or rather to compare what are the limitations and scopes of each study. The respective information was obtained via the project’s homepages, reports and interviews.

Information from HBM was linked with register information (environmental databases, perinatal health registers, chronic disease registries) and health indicators, and similarities and differences in data collection and data management between human biomonitoring studies and health examination surveys (HES) were evaluated. Based on the extracted data we generated an overview of indicators of environmental exposure and health, and correlated them with data on human biomarkers that are available for environmental health research. On this basis, we derived recommendations for future work towards integration of HBM in Health information (HI).

## Results

### State of the art of environmental health surveillance in Europe

In Europe, there are a number of countries that run cross-sectional HBM programmes or birth cohorts to monitor potential impacts of environmental chemicals on population health. The present inventory, builds on work performed by COPHES,^6^ EFSA [10], and WHO [11] as well as the work performed in the framework of ENRIECO [6] and CHICOS [5]. It includes most recent information collected in the course of BRIDGE Health.

National HBM programmes and birth cohorts cover thousands of samples from the healthy general adult population including breast-feeding mothers and new-born, as well as elderly women (50–65 years) and children aged 3–17 years. Questionnaire information covers residential environment, nutrition, smoking behaviour, socio-economic status, exposure-relevant life-style behaviour, and occupation. Sampled matrices are mainly urine and blood and substance groups covered comprise heavy metals, a broad range of organic pollutants (e.g. PCBs (polychlorinated diphenyls), PAHs (polycyclic aromatic hydrocarbons), PBDEs (polybrominated diphenyl ethers), PCDDs (polychlorinated dibenzo-p-dioxins), PCDFs (polychlorinated dibenzofurans), organochlorines, PFCs (perfluorocarbons), phthalates, herbicides, fungicides), VOCs (volatile organic compounds), markers for tobacco smoke and allergens.

### European cross-sectional HBM studies

Within Europe, several countries established cross-sectional HBM programmes to assess environmental pollution levels of their inhabitants as a baseline or in regular intervals. In addition, some MS or regions in the European Union (i.e. Flanders in Belgium, Czech Republic, and Slovenia) have established legislation for mandatory HBM programmes since the early years of 2000. The legal framework was set out due to chemical incidents and industrial pollution legacy. The main programmes existing in the European Union are highlighted and presented in the following. A more exhaustive table is available in the Additional file 1.

The German Environmental Survey (GerES) is a nationwide population representative study on human exposure to environmental chemicals and its sources. The first GerES started in 1985 and up to now, five GerES have been conducted. In conclusion, these five studies assessed a wide range of chemical exposure burden on adults, children and adolescents in Germany and specified reference values, that are valuable when it comes to policy making [12–15].

The Czech Environmental Health Monitoring System (CZ-HBM) measured various metals, PCBs, cotinine, and organochlorines in adults, breastfeeding primiparas, and children (age < 12) in 1994–2003, 2005–2009, 2009–2016 with 13,937 in each round [16].

The Flemish Environment and Health Study (FLEHS) has been running three cycles (2002 (4400 participants), 2007 (650 participants), and 2012 (120 mother child pairs) by now, and is currently performing its fourth cycle (started in 2017) [17, 18]. The French Nutrition and Health Survey (ENNS) started in 2006–2007 with a representative sample of around 4800 children and adults (age 3–74). A second round including children (age 6–17) and adults (age 18–74) took place in 2014–2015. ENNS analysed 42 chemicals in blood, urine and hair [19]. Furthermore, the ELFE^7^ [20] studies determined a broad range of environmental pollutants in children and adults.

Spain performed a representative overview of chemical exposure in the Spanish workforce. BIOAMBIENT.ES measured six different classes of environmental chemicals in 1892 participants aged 16 or older [21]. A new HBM survey focused on adolescents (age 14–16) has been initiated in 2017. This study named BEA (Biomonitorización En Adolescentes), studies the levels of metals and organic pollutants in samples of blood, urine and hair collected in 10 Spanish cities having more than 150,000 inhabitants.^8^ Slovenia performed a pilot survey from 2007 to 2009 with 320 breastfeeding mothers and their partners. In 2011 Slovenia started a follow-up HBM study with 960 participants [22].

Italy run a population study (PROBE) with adults aged 18–65 years to determine the exposure to 20 metals and a number of POPs on the healthy general population in 2008–2010 and 2011–2012 [23, 24].

In the Nordic countries Sweden has an environmental monitoring program including HBM and Norway is initiating a survey [25].

Besides these population representative programmes there are numerous smaller regional or research studies in other European countries, which are covering similar age groups and chemicals, as well as a European-wide HBM pilot performed among 1844 children and their mothers in 17 European countries (COPHES/DEMOCOPHES), that measured phthalate compounds, cadmium in urine, and mercury in hair [3] Table 1.

**Table 1:** Overview on European cross-sectional HBM studies

	Scientific Focus	Size	Age groups	Matrix	Chemicals	Frequency
Belgium FLEHS	Wide range of exposure burden in Flanders, reference values	650	new-borns and their mothers 14–15 y/o adults 20–40 y/o adults	Cord blood, blood, urine hair	metals, dioxins, PCBs, PAHs, dichlorodiphenyltrichloroethane, phthalates, musks, parabens and organophosphate pesticides, flame retardants	4× since 2002
Czech Republic EHMS	Core set of chemicals measured in the Czech population	1290	18–58 y/o adults 8–10 y/o children breastfeeding primiparas g	Blood urine, breastmilk, teeth	metals, PCBs, cotinine, organochlorines	Annually since 1994
France ENNS	Wide range of exposure burden on children and adults in France	4790	3–17 y/o children 18–74 y/o adults	Blood, urine hair	metals, PCBs, organochlorines, pyrethroids, organophosphates	Once, 2006–2007
Germany GerES	Wide range of exposure burden on adults, children and adolescents in Germany, reference values	1790–4822	adults: GerES I-III: 18–79 y children and adolescents: GerES III-V: 6–17 y (GerES III) 3–14 y (GerES IV) 3–17 y (GerES V) (in preparation: adults: GerES VI: 18–79 y)	Blood urine	metals, organochlorines, cotinine, organophosphates, chlorophenols, polycyclic aromatic hydrocarbons, pyrethroids, phthalates, bisphenol A, phenanthrenes, plasticiser substitutes, pyrrolidones	5× since 1985
Italy PROBE	POPs´ and metals’ internal dose in adults to highlight the environmental impact on the health of Italian population	1423	18–65 y/o adults	Blood	20 metals, POPs	2× (both 2008)
Slovenia HBM program	Pilot study for establishing reference values for selected chemicals in Slovenia	320	Breast feeding first time mothers (20–35 y/o) and their male partners	Blood breast milk	metals, PCBs, PBDEs, PCDDs, PCDFs	2× (2007, 2011)
Spain- BIOAMBI-ENT.ES	Estimation of levels of heavy metals and other substances on the Spanish active workforce	1892	Participants (≥16 y/o)	Blood, urine hair	metals, PCBs, cotinine, organochlorines, PAHs, PBDEs, perfluorinated alkyl substances, phthalates, DINCH	Once, 2009–2010
Spain BEA	Study of the adolescents to environmental pollutants and determinant factors	500	Adolescents (14–16 y/o)	Blood, urine, hair	Metals, bisphenols, triclosan, phthalates and others organic pollutants	Once, 2017
DEMOCOPHES	To test the feasibility of harmonized HBM in Europe	1844 (~ 240 per country)	Children 6–11 y/o and their mothers (≤ 45 y/o)	Hair urine	phthalates, cadmium, mercury	Once, 2010–2012

### European cohort studies

Over the last 25 years, a large number of pregnancy and birth cohorts have been established in Europe; some of them collected information on environmental exposures.

CHICOS^9^ covered a total of 77 European birth cohorts including more than 500,000 mother-child pairs [5]. ENRIECO^10^ included 37 of these 77 birth cohorts that collected information on environmental exposures including more than 350,000 study subjects [6].

The oldest cohort in the ENRIECO inventory started in 1985 whereas the youngest cohort covered by the inventory started in 2011 reflecting relatively contemporary environmental exposures across Europe. In all cohorts, biological samples were collected from the mother and/or the child and, more rarely, from the father. Sampling covered prenatal and postnatal periods and many types of tissue, including blood, urine, hair, nails, breast milk, placentas, and saliva. Outdoor air pollution, allergen exposures, passive smoking, and maternal occupation were assessed by many birth cohorts. Exposure areas that were less well covered in the cohorts included noise, ionizing and non-ionizing radiations, and chemical exposures such as brominated and fluorinated compounds, phthalates, and phenols. Twenty-seven cohorts measured or were measuring contaminants or their metabolites as markers of internal dose, mostly in the categories of metals, POPs, and tobacco smoke. A detailed description of each of these 37 birth cohorts and the environmental and health data collected is provided in Vrijheid et al. and Gehring et al. [6, 26].

**Fig. 1:**
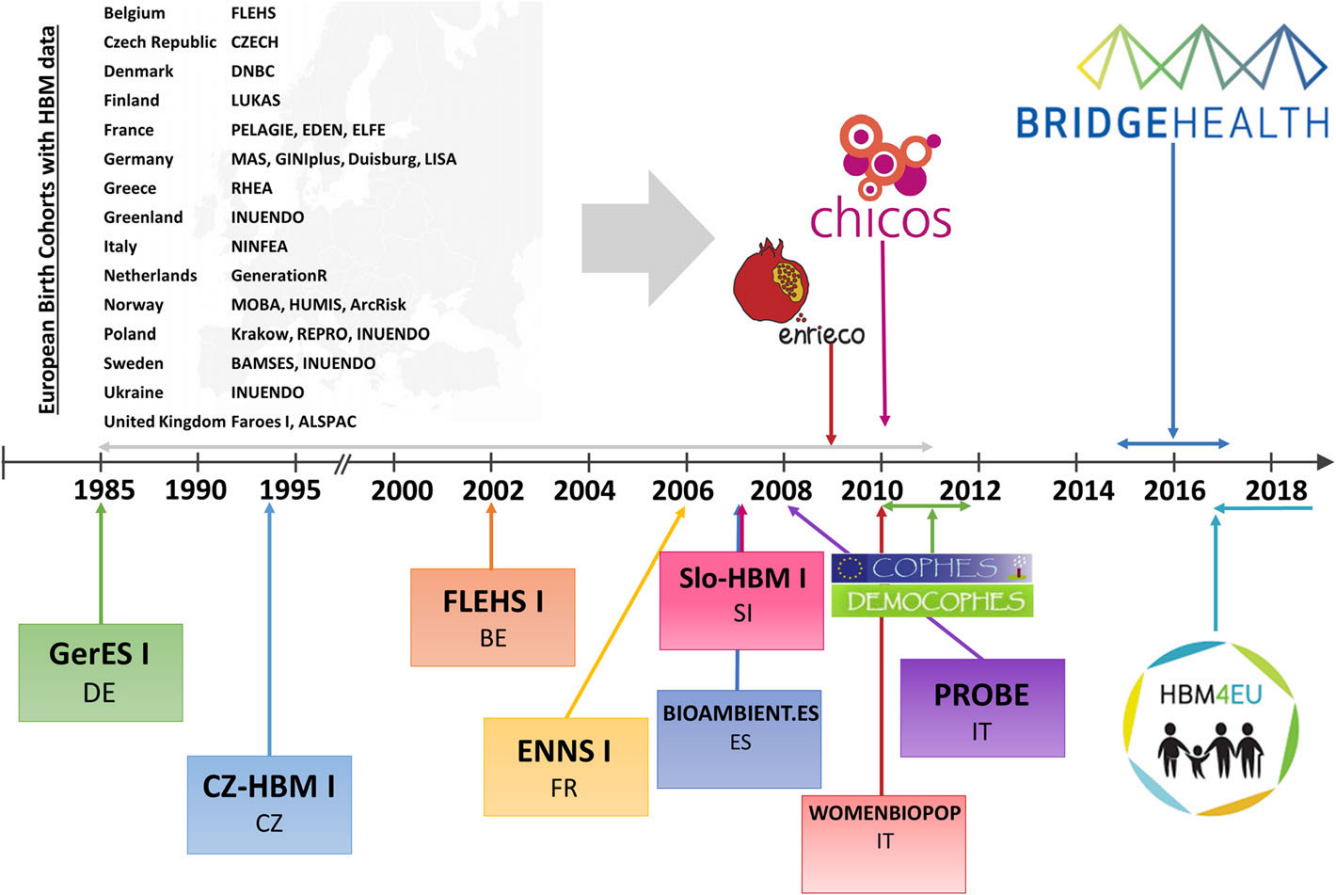
Starting dates of (only selected) European birth cohorts and HBM programmes (abbreviations), and related harmonisation efforts (logos)

Figure 1 presents a timeline with the beginning of large HBM studies within Europe. The timeline shows, that investigations of impacts of environmental chemicals (and other environmental stressors) on health started in the mid-1980s. Based and developed form worldwide and the previous European Union projects the European Joint Programme HBM4EU for the harmonization and further development of HBM started in 2017 in order to establish HBM as a prominent instrument to improve the policies on chemicals.

### Is it possible to link register information of environmental exposures and health indicators?

To interpret data from HBM surveys, and identify determinants of exposure and potential health effects at local, regional or national level, it is helpful if data can be matched with information from environmental and health registries. The environmental registries include European data sources such as INSPIRE and IPChem for environmental contamination, national databases in the case of drinking water, and the database of the European food safety agency (EFSA) for food items. Data related to health impacts (disease registries) are found either in national health registers [27], or at project level. Only registers for cancer and congenital anomalies are managed at European level by the Joint Research Centre JRC (ENCR-JRC, EUROCAT^11^) to date [3]. HBM-based indicators or data are available mainly in study databases but will be more and more integrated in IPChem within the currently running HBM4EU initiative. Table 2 gives a comprehensive overview on the most important environmental and health indicator data and/or relevant information within European programs and the corresponding human biomarkers nowadays available in Europe. By this it gets determinable, which indicators/data can be used in order to study either the human exposure route (human biomarkers, in columns), to study changes of the internal body burden from chemicals and which health indicators/data and environmental indicators are relevant to be linked to HBM biomarkers. A HBM-based indicator may include information about exposure routes, the occurrence of the substance in the environment or consumer good, the known health effects and finally the internal body burden of a certain population group. For example, Cotinine, a metabolite from nicotine, is a good and objective marker to assess exposure burden from tobacco smoke, and exposure from heavy metals in food to assess ‘*Exposure of children to chemical hazards in food’* could also be much better detected with HBM than with nutrition surveys. Additional indicators might be developed for other substances of very high concern.

**Table 2:** Overview of indicator data on environmental exposure, health and data of human biomarkers that are available for environmental health research

Indicators and/or relevant information within European programs	Biomarkers	POPs: dioxins furans, PCBs	Metals: lead, arsenic, mercury cadmium	Biocides: pesticides, herbicides fungicides	Flame retardants: polybrominated/fluorinated diphenyl ethers, organochlorines, organophosphates	Plasticizers/plastic compounds: (bis-) phenols, phthalates	Coatings: perfluorinated compounds	Personal care products: Polycyclic musks, disinfectants, trihalomethans, sunscreens	Smoking: Cotinine and metabolites	VOCs and VOCs: benzene, PAHs	Acrylamide & metabolites	Mycotoxins aflatoxin
**HBM EXPOSURE BIOMARKERS**												
**EXPOSURE ROUTE**												
**Outdoor air**												
Emission of acidifying substances, O_3_, prim.PM, sec.PM (EEA), PM_10_(ENHIS)										**x**		
Exceedance of air quality limit values in urban areas (EEA)										**x**		
Particulate matter exposure PM10 (ECHI55)			x							x		
**Indoor air**												
Indoor air (IPChem)		**x**	**x**		**x**	**x**	**x**		**x**	**x**		**x**
**Water**												
oxygen consuming substances (EEA) + nutrients in freshwater (EEA)				**x**								
contaminants in water (national databases)												
**Soil** (national databases)		**x**	**x**	**x**						**x**		
**Food**												
chemical contaminants: due to food production, distribution, packaging or consumption (EFSA)		**x**	**x**			**x**	**x**			**x**	**x**	
additives (EFSA)												
chemical residues: pesticides, hormones (EFSA)				**x**								
**Consumer goods**												
Consumer goods direct contact, other than food (REACH^1^)					**x**	**x**	**x**	**x**				
**Industrial emissions**												
pollutant release to air, water and waste from ind. Facilities (EEA)		**x**	**x**	**x**	**x**					**x**		**x**
**Waste**												
household consumption (EEA)			**x**	**x**								
diversion waste from landfill (EEA)			**x**	**x**						**x**		
**Occupation**												
work-related health risks (ECHI53)		**x**	**x**	**x**	**x**	**x**	**x**			**x**		**x**
**HEALTH INDICATORS/INFORMATION**												
**Smoking-related health outcomes**												
smoking-related deaths (ECHI15)									x			
regular smokers (ECHI44), pregnant women smoking (ECHI45)									x			
**Cardiovascular outcomes**												
blood pressure (ECHI43)			x						x	x		
**Cancer**												
cancer incidence (ECHI20)		x	x	x					x	x	x	
**Diabetes**												
diabetes:self-reported+register-based prevalence (ECHI21A and B)		x	x	x	x	x	x	x		x		
**Fertility**												
Time to pregnancy (HBM)		x	x	x	x	x	x	x				
total fertility rate (ECHI4)		x	x	x	x	x	x	x				
Sexual maturation (HBM)		x	x	x	x	x	x	x				
**Immunologic outcomes**												
asthma, self-reported +register-based prevalence (ECHI26A and B)		x	x	x	x	x	x	x	x	x	x	x
COPD, self-reported +register-based prevalence (ECHI27A and B)		x	x	x	x	x	x	x	x	x	x	
Allergies, allergic rhinitis, asthma, eczema, food allergy (HBM)		x	x	x	x	x	x	x	x	x	x	x
Infections (respiratory and others)		x	x	x	x	x	x	x	x	x	x	x
**Neurologic outcomes**												
dementia/Alzheimer (ECHI22)			x	x	x	x				x		
depression, self-reported +register-based prevalence (ECHI23A and B)			x	x	x	x				x		
cognitive tests (HBM)			x	x	x	x				x		
behaviour, autism, hyperactivity disorders (HBM)			x	x	x	x				x		
growth and obesity, metabolic syndrome (HBM)		x	x	x	x	x	x	x	x	x		
**Fetal, neonatal, and child health**												
Spontaneous abortion/stillbirths/terminations (HBM)		x	x	x	x				x	x		
Gestational age (HBM)		x	x	x	x				x	x		
(low) birth weight (ECHI28)		x	x	x	x	x			x	x	x	
mortality, birth weight, plurality (C1-C5)		x	x	x	x	x			x	x	x	
Prevalence +deaths of selected congenital anomalies (R1, R3)		x	x	x					x	x	x	
Distribution of Apgar score at 5 min (R2)		x	x	x	x	x	x	x	x	x	x	
Prevalence of cerebral palsy (R4)			x	x						x		
**Maternal health**												
Maternal mortality ratio (C6, C7)												
Incidence of severe maternal morbidity (R6)		x	x	x	x	x	x	x	x	x	x	
Mode of delivery (HBM)		x										
**Health in general**												
body mass index (ECHI42)		X			x	x	x	x	X			

Crosses in the table suggest which indicators/data can be linked in order to study either the human exposure route leading to measurable internal concentrations measured in humans (human biomarkers, in columns) and furthermore linking these biomarkers with health indicators/data (Note: EEA = Environmental Core Indicator; (ECHI = European Core Health indicators (ECHI nr); Health indicators of EUROPERISTAT (C = core, R = recommended); effect biomarkers of HBM studies/cohorts (HBM); IPCheM = Information Platform for Chemical Monitoring for inventarisation of indoor, HBM, food/feed and environmental data)

### Differences and options for synergies with health examination surveys (HES) – Are HBM and HES protocols compatible?

A Health Examination Survey (HES) collects health information from questionnaires and physical examinations. This covers objective measurements such as blood pressure, weight, height and the assessment of general blood and urine parameters. The European Health Examination Survey EHES^12^ works towards European wide harmonisation of HES.

The combination of HBM with a Health Examination survey enables the correlation of environmental exposure (internal body burden) and health status and contributes to a better understanding of the links between environmental exposure, lifestyles and health effects.

There are some successful examples of large-scale programs combining HBM, HES, and nutritional surveys or Health information surveys (HIS). The National Health and Nutrition Examination Survey of the United States of America (NHANES) combines a standardized physical examination in a mobile examination centre, with a home interview to assess participants’ health [28, 29] and collecting information on the body burden for a broad range of chemicals in random study population subgroups.

The Canadian Health Measure Survey (CHMS) uses a mobile examination centre to collect health parameters combined with a clinical questionnaire, and collects biological samples that are analysed for a broad range of environmental chemicals. Since the second Cycle of the CHMS, also indoor air and tap water are monitored [30].

The Korean National Health and Nutrition Examination Survey (KNHANES) was established to collect detailed information on health and nutrition status of Korean citizens. In 2007, the survey became a continuous, annual survey program. The survey is divided into three parts (health interview, health examination, and nutrition survey), and urine and blood samples are collected from participants aged 10 years and over. In 2005 the survey was combined with a human biomonitoring survey, Korean national survey for environmental pollutants in the human body (KorSEP), in order to add information on real body burdens [31, 32].

Within Europe, a number of EU member states such as Germany and France have established specific surveillance systems for health risks from environmental stressors, or have successfully combined HES, HIS with population based cross-sectional HBM programmes or longitudinal cohorts within the framework of their national environment and health action programmes (EHAPs).

In Germany, the German Environmental Survey (GerES) is linked to the German Health Interview and Examination Survey for Adults (DEGS) and for Children and Adolescents (KiGGS) performed by the Robert Koch Institute [33] All of the mentioned surveys collect comprehensive data on the health status of the respective age group. The surveys involve questionnaires, physical examinations and blood and urine collection for laboratory analysis in a subsample of the DEGS or KIGGS population. GerES performs human biomonitoring mostly of urine and blood samples, ambient monitoring of drinking water, house dust and indoor air and collects information on possible exposure pathways and living conditions via questionnaires [34]. The data sets of both surveys can be used in combination.

The French Étude Nationale Nutrition Santé (ENNS) is combining health and nutrition studies (information on food consumption, nutritional status, and physical activity) in the general population in France with measurements of nutritional and environmental pollutants (HBM) in blood and urine [19]. Scandinavian countries have successfully combined HIS and birth cohorts, using nationwide data on social, environmental, and health registers for research purpose (i.e. Danish National Birth Cohort and the Norwegian Mother and Child Cohort).

There are some similarities and differences in data collection and study population between HBM and EHES Protocols. Most important differences cover sample size, age groups covered, questionnaire topics, tested markers, and sample analysis. Minor differences exist as regards recruitment, questionnaire design, interview type, examination site, sampled matrices, sampling, transport, and storage. Training, communication, ethics, and data protection rules apply similarly to both types of surveys. Table 3 provides an overview about major study design aspects.

**Table 3:** Important parameter in HBM and HES study design

Key parameter	HBM studies	HES (according to EHES^a^)
Study population	General adult population: Adults (18–79 years) Specific population/vulnerable groups: Children and adolescents (3–17 years) Breastfeeding mothers Mothers and their new born Older women 50–65 years	General adult population: 500 of each of the eight age-sex domains (25–34, 35–44, 45–54 and 55–64 years)
Questionnaire topics	Residential environment and residence Nutrition (with focus on exposure related food intake) Smoking behaviour Exposure-relevant behaviour (mother’s) occupation Socio-demography and socio-economic status	Based on the EHIS. Health status (self-perceived, chronic morbidity, activity limitations) Health care (hospitalisation) Health determinants (height, weight, physical activity) Background variables (education, labour status)
Sample matrix^b^	Blood (10/11) Urine (8/11) Scalp hair (4/11) Breast milk (2/11) Cord blood (1/11) Teeth (1/11)	Blood Urine
Tested markers	Heavy Metals: Cd, Pb, Hg Manmade chemicals (PCBs, PAHs, PBDEs, PCDDs, PCDFs, organochlorine, VOCs, PFCs, Phthalate, herbicide, fungicide) Biological: IgE, Cotinine etc.	Biological Glucose Total cholesterol HDL Anthropometrical Weight Height Blood pressure Waist circumference

^a^ http://www.ehes.info/

^b^Sample matrix data assessed by Table 4 of Supplemental material

## Discussion

### State of the art of environmental health surveillance in Europe

Although cross-sectional HBM programmes and birth cohorts differ in target population, sampling scheme, recruitment, collection of biological matrices, and the number and types of chemicals analysed, they both contribute important information about impacts of chemicals and other environmental stressors on health.

The inventory on European HBM studies and birth cohorts shows clear that similar chemical groups and biological matrices are examined in cross sectional and longitudinal large or small-scale studies.

Estimates of exposure to other environmental stressors such as noise and PM, are usually obtained using geographical information systems (GIS) applications and available for a vast majority of the population and therefore highly suitable for environmental health surveillance. Recently omics technology is coming up, in assessing exposure-related changes in biological molecules (transcriptomics, epigenomics, metabolomics, proteomics) exposome is hardly used in environmental health surveillance.

### Is it possible to link register information of environmental exposures and health indicators?

From the comprehensive Table 2 it can be seen that within Europe, different EU institutions and reporting schemes gather a wealth of information on pollutants in air, soil, water, and/or food, and on diseases incidence/prevalence.

There are a considerable number of indicators available that collect information on health, such as the WHO European Region ENHIS (Environment and health information system) list and the European ECHI (European Core Health Indicators).

Indicators for environmental exposure cover the fields of nutrition, air pollution, noise, indoor environment, temperature, and outdoor pollution (EEA core indicators).

There are quite a number of associations between heavy metals, biocides, flame retardants, plasticizers, chemicals in personal care products and VOCs, with indoor air, food safety, consumer goods, and work-related health risks, and there are broad association between the vast majority of chemical groups and health indicators or health end points such as diabetes and other metabolic disorders [35], with allergies, COPD, neurological disorders, and perinatal health [36], as well as association between certain chemical and cancer [37, 38], which are not reflected in HI yet. This is for example true for heavy metals, which concentrations get determined routinely, when indoor air- or water quality is measured. However, the fact that heavy metals are also related to various health outcomes like for example, diabetes or neurologic outcomes, is not acknowledged by any available database. The other way around, when looked at health outcomes in the case of diabetes, there is also no link between this NCD and several stressors like POPs, the mentioned heavy metals, and plastic compounds..

Hence there is a need to integrate environmental health research and surveillance into the EU Health information system. To improve surveillance and monitoring strategies, the field of indicators are coming to the fore. Indicators are tools to detect positive or negative developments in different fields of actions, such as environmental protection or sustainability and can highlight success or failure of actions that have been taken. Consequently, indicators are used as a control instrument that delivers information about the progress that has been made in reaching special goals (e.g. to minimize exposure burden from environmental chemicals in the European population).

But indicators are not commonly linked to each other. This publication points out, that there is a great potential in the linking of indicators of various fields (chemical exposure, health outcomes) in terms of determining environmental health in a more adequate manner. This is of importance when it comes to the transformation process of science into policies, as only a valid presentation of chemical exposure in terms by indicators is suitable for the definition of legally enforceable and binding health-based guidance value reference values.

### Differences and options synergies with health examination surveys (HES)

The direct comparison of HBM- and HES-studies show, that there are a lot of similarities in the fieldwork part of the study design, in sampling, data management, quality and ethical issues, so that despite differences in the study population, questionnaire, matrix and chemical analysis, it should be possible to combine HES, chemical biomarker assessment in humans not only at national but also at European level, if certain adaptations are made. To our knowledge, this publication represents the first direct comparison of HBM- and HES-parameters. As already elaborated upon, there are great synergies between those two study types, which can have, if used in appropriate means, a large added value in impacting environmental health policy making.

## Conclusions

Given the wealth of information that HBM may provide on environmental determinants for health and the lack of tools to report on it at the level of the European Union, there is an urgent need to integrate environmental health research and surveillance into an EU Health information system, and into EU public health and consumer protection policies.

The current inconsistencies in data collections in the existing databases and reporting schemes for HBM, environmental, nutrition and disease registries strongly hamper the usability of data. However, there is considerable perspective to align data collection. in terms of geographical stratification, temporal changes, information on life-style, potential exposure and socio-economic risks.

Regarding HBM and HES surveys there are also considerable differences but at the same time they can be performed together, improving its cost-efficiency. There is a need for considerable further efforts to generate appropriate indicators for monitoring exposure burden levels to environmental chemicals. Aside from the indicator data collected within the EU institutions, it would be advisable to have additional EU wide indicators in the field of: (i) environmental health: e.g. HBM biomarkers, occupational groups, noise level (in cities), environments, (heavy) traffic density, as well as (ii) health: allergy, infections, growth, behaviour data, spontaneous abortions.

Based on these conclusions it can be recommended to:

Include environmental health information in a European Health information system (HIS) in order to meet the requirements of the Agenda 2030, and its SDG3, and to integrate HBM-based indicators for environmental health on existing lists, like ECHI.

A first step towards this goal should be a better alignment of health, environmental health, environmental, and administrative data and data collections and bases, as well as of HES survey design with HBM surveys and cohorts. For now, a first step would be to evaluate the data sets available on European level, combining all the current indicators/biomarker, as suggested in Table 2. Combining the information offers the possibility to create a complete European Environmental Health surveillance and warning system. Indeed, trends in these indicators and exceedances above health-based guidance values allow estimating or identifying (upcoming) potential health risks.

The alignment of HBM’s and HES´ study designs includes the adaption of the target populations, the questionnaires, and the chemical analysis. HES should be expanded to cover also children, adolescents, and new-borns with their mothers in the surveys. The questionnaire should comprise questions about potential exposure sources. The sampling could be expanded to cover hair and milk, and the chemical analysis should comprise at least some priority chemicals. Whereby the acquired personal data needs to be treated based on the new General Data Protection Regulation (GDPR) to be implemented as of May 25th, 2018 will require further harmonization on a European level. Whilst further work on HBM specific issues such as PBPK models, derivation of health based guidance values, development of HBM-based indicators, combination of HBM- and Health-Studies etc. will be performed in the European Human Biomonitoring Initiative HBM4EU,^13^ Health information specific topics such as integration of HBM relevant information in health and patient data registries allowing to trace back to occupation, life style, exposure or socio-economic status, or indicator development should be covered by any joint action or research infrastructure on Health Information, as a follow-up to BRIDGE Health in close cooperation with HBM4EU.

## Additional file

Additional file 1: **Table S4.** Overview of the large-scale national human biomonitoring surveys (sorted in chronological order from oldest to newest) in Europe and worldwide and their key aspects. (DOCX 21 kb)

## Abbreviations

BEA: Biomonitorización en adolescentes
BIOAMBIENT.ES: Representative overview of chemical exposure in the Spanish workforce
BMI: Body mass index
BRIDGE Health: BRidging information and data generation for evidence-based health policy and research
CHICOS: Child cohort research strategy for Europe
CHMS: Canadian health measure survey
CO: Carbon monoxide
COPD: Chronic obstructive pulmonary disease
COPHES: Consortium to perform human biomonitoring on a European scale
CZ-HBM: Czech environmental health monitoring system
DDE: Dichlorophenyldichloroethylene
DDT: Dichlorodiphenyltrichloroethane
DEGS: German health interview and examination survey for adults
DEMOCOPHES: DEMOnstration of consortium to perform human biomonitoring on a European scale
DINCH: 1, 2-Cyclohexane dicarboxylic acid diisononyl ester
EC: European commission
ECHI: European core health indicators
EEA: European environmental agency
EFSA: European food safety agency
EHAP: Environmental health action plans
EHMS: Environmental health monitoring system
EHS: Environmental health surveillance
ELFE: Etude longitudinale française depuis l’enfance
ENHIS: Environment and health information system
ENNS: French nutrition and health survey
ENRIECO: Environmental health risks in European birth cohorts
EU: European Union
EUROCAT: European surveillance of congenital anomalies
EUROPERISTAT: European perinatal health indicator
FLEHS: Flemish environment and health study
GerES: German environmental survey
GIS: Geographical information systems
H2020: Horizon 2020
HBGV: Health-based guidance values
HBM: Human biomonitoring
HBM4EU: European human biomonitoring initiative
HCB: Hexachlorobenzene
HDL: High density lipoprotein
HES: Health examination survey
HI: Health information
HiAP: Health in all policies
HIREP ERIC: European research infrastructure consortium on health information for research and evidence-based
HIS: Health information surveys
HIS: Health information system
IgE: Immunoglobulin E
INSPIRE: Infrastructure for spatial information in the European
IPChem: Information platform for chemical monitoring
KiGGS: German health interview and examination survey for children and adolescents
KNHANES: Korean National health and nutrition examination survey
MBT: Mercaptobenzothiazole
MoBa: Norwegian mother and child cohort study (MoBa)
NCD: Non-communicable diseases
NHANES: National health and nutrition examination survey of the United States of America
NO_2_: Nitrogendioxide
PAH: Polycyclic aromatic hydrocarbon
PBDE: Polybrominated diphenyl ethers
PBPK: Physiologically based pharmacokinetic
PCBs: Polychlorinated diphenyls
PCDDs: Polychlorinated dibenzo-p-dioxins
PCDFs: Polychlorinated dibenzofurans
PFCs: Perfluorocarbons
PM: Particulate matter
POPs: Persistent organic pollutants
SDG: Sustainable developmental goals
SHS: Second hand smoke
SO_2_: Sulfurdioxide
VOCS: Volatile organic compound(s)
WHO: World health organization

## Footnotes

1 WHO: http://data.euro.who.int/eceh-enhis/Default2.aspx?indicator_id=19

2 European Commission: https://ec.europa.eu/health/indicators/echi/list_en

3 (http://www.eu-hbm.info/cophes)

4 http://www.enrieco.org/

5
www.HBM4EU.eu

6
http://www.eu-hbm.info/cophes/a-special-issue-201charmonized-human-biomonitoring-on-a-european-scale-experiences-in-seventeen-countries201d-has-been-issued-in-august-2015-in-environmental-research

7
https://www.elfe-france.fr/en/

8 (http://bea.blogs.isciii.es/).

9
www.birthcohorts.net

10
www.enrieco.dk

11 http://www.eurocat-network.eu/

12 http://www.ehes.info/

13 https://www.hbm4eu.eu/

## References

[1] Kramers PG (2003). The ECHI project: health indicators for the European Community. Eur J Pub Health.

[2] EC European Commission The European parliament and the European Economic and Social Committee Brussels (2004). European environment and health action plan for 2004–2010 volume I and II.

[3] Den Hond E, Govarts E, Willems H, Smolders R, Casteleyn L, Kolossa-Gehring M, Schwedler G, Seiwert M, Fiddicke U, Castano A, Esteban M, Angerer J, Koch HM, Schindler BK, Sepai O, Exley K, Bloemen L, Horvat M, Knudsen LE, Joas A, Joas R, Biot P, Aerts D, Koppen G, Katsonouri A, Hadjipanayis A, Krskova A, Maly M, Morck TA, Rudnai P, Kozepesy S, Mulcahy M, Mannion R, Gutleb AC, Fischer ME, Ligocka D, Jakubowski M, Reis MF, Namorado S, Gurzau AE, Lupsa IR, Halzlova K, Jajcaj M, Mazej D, Tratnik JS, Lopez A, Lopez E, Berglund M, Larsson K, Lehmann A, Crettaz P, Schoeters G (2015). First steps toward harmonized human biomonitoring in Europe: demonstration project to perform human biomonitoring on a European scale. Environ Health Perspect.

[4] Schwedler G, Seiwert M, Fiddicke U, Issleb S, Holzer J, Nendza J, Wilhelm M, Wittsiepe J, Koch HM, Schindler BK, Goen T, Hildebrand J, Joas R, Joas A, Casteleyn L, Angerer J, Castano A, Esteban M, Schoeters G, Den Hond E, Sepai O, Exley K, Bloemen L, Knudsen LE, Kolossa-Gehring M (2017). Human biomonitoring pilot study DEMOCOPHES in Germany: contribution to a harmonized European approach. Int J Hyg Environ Health.

[5] Larsen PS, Kamper-Jorgensen M, Adamson A, Barros H, Bonde JP, Brescianini S, Brophy S, Casas M, Charles MA, Devereux G, Eggesbo M, Fantini MP, Frey U, Gehring U, Grazuleviciene R, Henriksen TB, Hertz-Picciotto I, Heude B, Hryhorczuk DO, Inskip H, Jaddoe VW, Lawlor DA, Ludvigsson J, Kelleher C, Kiess W, Koletzko B, Kuehni CE, Kull I, Kyhl HB, Magnus P, Momas I, Murray D, Pekkanen J, Polanska K, Porta D, Poulsen G, Richiardi L, Roeleveld N, Skovgaard AM, Sram RJ, Strandberg-Larsen K, Thijs C, Van Eijsden M, Wright J, Vrijheid M, Andersen AM (2013). Pregnancy and birth cohort resources in europe: a large opportunity for aetiological child health research. Paediatr Perinat Epidemiol.

[6] Vrijheid M, Casas M, Bergstrom A, Carmichael A, Cordier S, Eggesbo M, Eller E, Fantini MP, Fernandez MF, Fernandez-Somoano A, Gehring U, Grazuleviciene R, Hohmann C, Karvonen AM, Keil T, Kogevinas M, Koppen G, Kramer U, Kuehni CE, Magnus P, Majewska R, Andersen AM, Patelarou E, Petersen MS, Pierik FH, Polanska K, Porta D, Richiardi L, Santos AC, Slama R, Sram RJ, Thijs C, Tischer C, Toft G, Trnovec T, Vandentorren S, Vrijkotte TG, Wilhelm M, Wright J, Nieuwenhuijsen M (2012). European birth cohorts for environmental health research. Environ Health Perspect.

[7] Casas M, Cordier S, Martinez D, Barros H, Bonde JP, Burdorf A, Costet N, Dos Santos AC, Danileviciute A, Eggesbo M, Fernandez M, Fevotte J, Garcia AM, Grazuleviciene R, Hallner E, Hanke W, Kogevinas M, Kull I, Stemann Larsen P, Melaki V, Monfort C, Nordby KC, Nybo Andersen AM, Patelarou E, Polanska K, Richiardi L, Santa Marina L, Snijder C, Tardon A, van Eijsden M, Vrijkotte TG, Zugna D, Nieuwenhuijsen M, Vrijheid M (2015). Maternal occupation during pregnancy, birth weight, and length of gestation: combined analysis of 13 European birth cohorts. Scand J Work Environ Health.

[8] Gascon M, Sunyer J, Casas M, Martinez D, Ballester F, Basterrechea M, Bonde JP, Chatzi L, Chevrier C, Eggesbo M, Esplugues A, Govarts E, Hannu K, Ibarluzea J, Kasper-Sonnenberg M, Klumper C, Koppen G, Nieuwenhuijsen MJ, Palkovicova L, Pele F, Polder A, Schoeters G, Torrent M, Trnovec T, Vassilaki M, Vrijheid M (2014). Prenatal exposure to DDE and PCB 153 and respiratory health in early childhood: a meta-analysis. Epidemiology.

[9] Govarts E, Nieuwenhuijsen M, Schoeters G, Ballester F, Bloemen K, de Boer M, Chevrier C, Eggesbo M, Guxens M, Kramer U, Legler J, Martinez D, Palkovicova L, Patelarou E, Ranft U, Rautio A, Petersen MS, Slama R, Stigum H, Toft G, Trnovec T, Vandentorren S, Weihe P, Kuperus NW, Wilhelm M, Wittsiepe J, Bonde JP, Obelix and Enrieco (2012). Birth weight and prenatal exposure to polychlorinated biphenyls (PCBs) and dichlorodiphenyldichloroethylene (DDE): a meta-analysis within 12 European birth cohorts. Environ Health Perspect.

[10] Choi J., Mørck T.A., Polcher A., Knudsen L. E. and Joas A. 2014. EFSA supporting publications 2015:EN-724. [321 pp.]. Retrieved from http://www.efsa.europa.eu/publications.

[11] WHO 2015. Human biomonitoring: facts and figures. Retrieved from http://www.euro.who.int/__data/assets/pdf_file/0020/276311/Human-biomonitoring-facts-figures-en.pdf.

[12] Kolossa-Gehring M., Becker K., Conrad, A., Schröter-Kermani C., Schulz C. and Seiwert M (2012): Health-related Environmental Monitoring in Germany: German Environmental Survey (GerES) and Environmental Specimen Bank (ESB), . Biomarkers and Human Biomonitoring, 16. 10.1016/j.ijheh.2011.10.013 22172995

[13] Kolossa-Gehring M, Fiddicke U, Leng G, Angerer J, Wolz B (2017). New human biomonitoring methods for chemicals of concern-the German approach to enhance relevance. Int J Hyg Environ Health.

[14] Schulz C, Conrad A, Becker K, Kolossa-Gehring M, Seiwert M, Seifert B (2007). Twenty years of the German environmental survey (GerES): human biomonitoring--temporal and spatial (West Germany/East Germany) differences in population exposure. Int J Hyg Environ Health.

[15] Schulz C, Kolossa-Gehring M, Gies A (2017). German environmental survey for children and adolescents 2014-2017 (GerES V) - the environ-mental module of wave 2 of the KiGGS study. Journal of Health Monitoring.

[16] Cerna M, Krskova A, Cejchanova M, Spevackova V (2012). Human biomonitoring in the Czech Republic: an overview. Int J Hyg Environ Health.

[17] Schoeters G, Colles A, Den Hond E, Croes K, Vrijens J, Baeyens W, Nelen V, Van de Mieroop E, Covaci A, Bruckers L, Van Larebeke N, Sioen I, Morrens B, Loots I (2012). The Flemish environment and health study (FLEHS) – second survey (2007–2011): establishing reference values for biomarkers of exposure in the Flemish population. Biomarkers and human biomonitoring (Ed.), issues in toxicology, no. 9 (Vol. volume 1: ongoing programs and exposures, chap. 2F): Royal Society of Chemistry.

[18] Schoeters G, Govarts E, Bruckers L, Den Hond E, Nelen V, De Henauw S, Sioen I, Nawrot TS, Plusquin M, Vriens A, Covaci A, Loots I, Morrens B, Coertjens D, Van Larebeke N, De Craemer S, Croes K, Lambrechts N, Colles A, Baeyens W (2017). Three cycles of human biomonitoring in Flanders - time trends observed in the Flemish environment and health study. Int J Hyg Environ Health.

[19] Fréry N, Stéphanie V, Anne E, Clémence F (2012). Highlights of recent studies and future plans for the French human biomonitoring (HBM) programme. Int J Hyg Environ Health.

[20] Esteban M (2017). Équipe de surveillance et d’épidémiologie nutritionnelle (Esen). Étude de santé sur l’environnement, la biosurveillance, l’activité physique et la nutrition (Esteban), 2014–2016. Santé publique France, 42.

[21] Pérez-Gómez B, Pastor-Barriuso R, Cervantes-Amat M, Esteban M, Ruiz-Moraga M, Aragonés N, Pollán M, Navarro C, Calvo E, Román J, López-Abente G, Castaño A (2013). BIOAMBIENT.ES study protocol: rationale and design of a cross-sectional human biomonitoring survey in Spain. Environ Sci Pollut Res Int.

[22] Perharic L, Vracko P (2012). Development of national human biomonitoring programme in Slovenia. Int J Hyg Environ Health.

[23] Alimonti A., Bocca B., Mattei D. and Pino A. 2011. Programme for biomonitoring the Italian population exposure (PROBE): internal dose of metals. Retrieved from www.iss.it/binary/publ/cont/11_9_web.pdf.

[24] De Felip E, Abballe A, Albano FL, Battista T, Carraro V, Conversano M, Franchini S, Giambanco L, Iacovella N, Ingelido AM, Maiorana A, Maneschi F, Marra V, Mercurio A, Nale R, Nucci B, Panella V, Pirola F, Porpora MG, Procopio E, Suma N, Valentini S, Valsenti L, Vecchie V (2015). Current exposure of Italian women of reproductive age to PFOS and PFOA: a human biomonitoring study. Chemosphere.

[25] Knudsen LE. and Hansen PW., 2017. Nordic workshop for scientists and regulatory agencies discussing HBM4EU - The Human Bio-monitoring Initiative. Paper presented at the Nordisk Ministerråd, Copenhagen. Retrieved from http://norden.diva-portal.org/smash/record.jsf?pid=diva2%3A1121454&dswid=-5050.

[26] Gehring U, Casas M, Brunekreef B, Bergstrom A, Bonde JP, Botton J, Chevrier C, Cordier S, Heinrich J, Hohmann C, Keil T, Sunyer J, Tischer CG, Toft G, Wickman M, Vrijheid M, Nieuwenhuijsen M (2013). Environmental exposure assessment in European birth cohorts: results from the ENRIECO project. Environ Health.

[27] Thygesen LC, Ersboll AK (2011). Danish population-based registers for public health and health-related welfare research: introduction to the supplement. Scand J Public Health.

[28] Calafat AM, Needham LL (2009). What additional factors beyond state-of-the-art analytical methods are needed for optimal generation and interpretation of biomonitoring data?. Environ Health Perspect.

[29] Calafat Antonia M (2012). The U.S. National Health and nutrition examination survey and human exposure to environmental chemicals. Int J Hyg Environ Health.

[30] Health Canada 2015. Third Report on Human Biomonitoring of Environmental Chemicals in Canada - Results of the Canadian Health Measures Survey Cycle 3 (2012–2013). Retrieved from http://www.hc-sc.gc.ca/ewh-semt/pubs/contaminants/chms-ecms-cycle3/index-eng.php.

[31] Ha M, Kwon HJ, Leem JH, Kim HC, Lee KJ, Park I, Lim YW, Lee JH, Kim Y, Seo JH, Hong SJ, Choi YH, Yu J, Kim J, Yu SD, Lee BE (2014). Korean environmental health survey in children and adolescents (KorEHS-C): survey design and pilot study results on selected exposure biomarkers. Int J Hyg Environ Health.

[32] Kim Y (2014). The Korea National Health and nutrition examination survey (KNHANES): current status and challenges. Epidemiol Health.

[33] Kurth B-M, Panagiotis K, Heike H, Martin S, Rudiger D, Ute E, Heidrun K, Hiltraud K, Michael L, Gert M, Hannelore N, Angelika R, Christa S-N, Liane S, Robert S, Heribert S, Michael T, Wulf T, Ute W (2008). The challenge of comprehensively mapping children's health in a nation-wide health survey: Design of the German KiGGS-study. BMC Public Health.

[34] Schulz C, Seiwert M, Babisch W, Becker K, Conrad A, Szewzyk R, Kolossa-Gehring M (2012). Overview of the study design, participation and field work of the German environmental survey on children 2003-2006 (GerES IV). Int J Hyg Environ Health.

[35] Thayer KA, Heindel JJ, Bucher JR, Gallo MA (2012). Role of environmental chemicals in diabetes and obesity: a National Toxicology Program workshop review. Environ Health Perspect.

[36] Vrijheid M, Casas M, Gascon M, Valvi D, Nieuwenhuijsen M (2016). Environmental pollutants and child health-a review of recent concerns. Int J Hyg Environ Health.

[37] Vineis P, Wild CP (2014). Global cancer patterns: causes and prevention. Lancet.

[38] Ward EM, Schulte PA, Straif K, Hopf NB, Caldwell JC, Carreon T, DeMarini DM, Fowler BA, Goldstein BD, Hemminki K, Hines CJ, Pursiainen KH, Kuempel E, Lewtas J, Lunn RM, Lynge E, McElvenny DM, Muhle H, Nakajima T, Robertson LW, Rothman N, Ruder AM, Schubauer-Berigan MK, Siemiatycki J, Silverman D, Smith MT, Sorahan T, Steenland K, Stevens RG, Vineis P, Zahm SH, Zeise L, Cogliano VJ (2010). Research recommendations for selected IARC-classified agents. Environ Health Perspect.

