# Supplementary material for: Environmental health surveillance in a future European health information system
Source: Arch Public Health. 2018 Jun 28;76:27. doi: 10.1186/s13690-018-0272-6 (PMC6022511; doi:10.1186/s13690-018-0272-6)
Supplement: Supplementary file 1 — Table S4. Overview of the large-scale national human biomonitoring surveys (sorted in chronological order from oldest to newest) in Europe and worldwide and their key aspects. (DOCX 21 kb) [file 13690_2018_272_MOESM1_ESM.docx]

Table s4: Overview of the large-scale national human biomonitoring surveys (sorted in chronological order from oldest to newest) in Europe and worldwide and their key aspects

|  | **Scientific Focus** | **Population size** | | **Age group** | **Matrix** | **Number of Chemicals** | **Sampling locations across the country** | **Recruitment** | **Questionnaire** | **Representative for** | **Sampling period** | **Frequency of the HBM program** | **Measures in food or water** |
| --- | --- | --- | --- | --- | --- | --- | --- | --- | --- | --- | --- | --- | --- |
| Member States of the EU | | | | | | | | | | | | | |
| Belgium - FLEHS | Wide range of exposure burden in Flanders, reference values | 650 | Three age groups: newborns and their mothers, 14-15 years old adolescents and 20-40 years old adults | | Cord blood, blood, urine and hair | >40 | 5, including 2 hot-spot areas | Maternities, schools and provincial institutes | Self-administered | Respective population groups of Flanders | 2007-2011 | 3 surveys since 2002 | No |
| Czech Republic – EHMS6 | Core set of chemicals measured in the Czech population | 1,290 | Three age groups: adults (blood donors aged 18-58 years), children aged 8-10 years, and breastfeeding primiparas g | | Blood and urine, breastmilk, teeth | 6 | 4 urban/  suburban areas | Blood donors via Transfusion Department, children via schools, breastfeeding women via maternity clinic | Face-to-face interview | Respective Czech population (8‑10; 18-58 years old) | 1994-2003 | Annually since 1994 | Yes |
| France – ENNS^4^ | Wide range of exposure burden on children and adults in France | 4,790 | Children 3‑ 17 years old and adults 18-74 years old | | Blood, urine and hair | 42 | 190 | Households via phone user databases | Face-to-face interview and self-administered | Children and adults in France (3‑74 years old) | 2006-2007 | Once | No |
| Germany –  GerES (I-V) | Wide range of exposure burden on adults, children and adolescents in Germany, reference values | 1,790-4822 | adults: GerES I-III:  18-79 y  children and adolescents: GerES III-V:  6-17 y (GerES III) 3-14 y (GerES IV)  3-17 y (GerES V)  (in preparation: adults: GerES VI: 18-79 y) | | Blood and urine | For GerES V:  ̴ 80 (inter alia metals, organochlorines, cotinine, chlorophenols, polycyclic aromatic hydro-carbons and naphthalene, polychlorinated biphenyls, perfluorocarboxylic, perfluorosulfonic acids, phthalates, plasticiser subsitutents, bisphenol A, triclosan, parabens, pyrrolidones) | 167 | population register | Face-to-face interview | the respective age group in Germany | GerES I: 1985-1986; GerES II: 1990-1992; GerES III: 1997-1999; GerES IV: 2003-2006; GerES V: 2014-2017 | 5 surveys since 1985 | Yes |
| Italy - PROBE | Metals’ internal dose in  adults to highlight the environmental impact on the health of Italian population | 1,423 | Adults  (18-65 y/o) | | Blood | Metals | 5 urban areas | Pre-determined sampling units | Face-to-face interview | Adult Italian population (18-65 y/o) living in urban areas | 2008-2010 | Once | No |
| Slovenia – national HBM program | Pilot study for establishing reference values for selected chemicals in Slovenia | 320 | Breast feeding first time mothers (20-35 years old) and their male partners | | Blood and breast milk | 10 substance groups | 4 unpolluted rural areas | Prenatal clinics | Self-administered | Respective Breast-feeding mothers and their partners | 2007-2009 | Once | No |
| Spain- BIOAMBI-ENT.ES | Estimation of levels of heavy metals, POPs, and other substances on the Spanish active workforce | 1,892 | Participants  (≥16 y/o) | | Blood, Urine and hair | 43 | nationwide | Randomly selected sampling units | Self-administered | Population in Spain  ≥16 y/o | 2009-2010 | Once | No |
| Spain - BEA | Study of the adolescents to environmental pollutants and determinant factors | 500 | Adolescents (14-16 y/o) | | Blood, urine and hair | > 13 | 10 big cities | High schools | Self-administered | Urban adolescents of main autonomous communities of Spain | Sep 2017 – Feb 2018 | Once | No |
| EU-Wide | | | | | | | | | | | | | |
| Europe – DEMOCOPHES^7^ | To test the feasibility of harmonized HBM in Europe | 1,844 (~240 per country) | | Children 6‑11 years old and their respective mothers (up to 45 years old) | Hair and urine | 5 | 17 EU countries, rural and urban per country | 4 via population registries and 13 via schools | Face-to-face interview | children and their mothers in Europe (6‑11 and mothers up to 45 years old) | 2011-2012 | Once | No |
| Out of EU | | | | | | | | | | | | | |
| USA – NHANES | Wide range of exposure burden on children and adults in USA, reference values | ~5,000 | | Children >1-year, pregnant women, one third is 6 years or 12 years and older, adults all ages | Blood and urine | 219 | 30 | population register | Face-to-face interview | Respective children and adults in USA | One year | Annually since 1999 | Yes |
| CANADA – CHMS Cycle 2 | Wide range of exposure burden on the Canadian population, reference values | 5,800 | | Children and adults 3-79 years old | Blood and urine | 48 | 16 sites across Canada | population register | Face-to-face interview | Respective Canadian population (3‑79 years old) | 2012-2013 | Every two years | Yes |
| South Korea - KorSEP III | Exposure burden on Korean adults | 5,087 | | Adults aged 20 or older | Blood and urine | 19 | 193 areas in South Korea | Sampling units selected (20 households per sampling unit) | Face-to-face interview | Adults in South Korea (20 years and older) | 2008 | 3 surveys since 2005 | No |
